# Analysis of somatic mutations identifies signs of selection during in vitro aging of primary dermal fibroblasts

**DOI:** 10.1111/acel.13010

**Published:** 2019-08-05

**Authors:** Narisu Narisu, Rebecca Rothwell, Peter Vrtačnik, Sofía Rodríguez, John Didion, Sebastian Zöllner, Michael R. Erdos, Francis S. Collins, Maria Eriksson

**Affiliations:** ^1^ National Human Genome Research Institute National Institutes of Health Bethesda MD USA; ^2^ Department of Biostatistics University of Michigan Ann Arbor MI USA; ^3^ Department of Biosciences and Nutrition, Center for Innovative Medicine Karolinska Institutet Huddinge Sweden; ^4^ Department of Psychiatry University of Michigan Ann Arbor MI USA

**Keywords:** aging cell, cell mosaicism, genome instability, molecular biology of aging, positive selection, somatic mutation, tissue heterogeneity

## Abstract

Somatic mutations are critical for cancer development and may play a role in age‐related functional decline. Here, we used deep sequencing to analyze the prevalence of somatic mutations during in vitro cell aging. Primary dermal fibroblasts from healthy subjects of young and advanced age, from Hutchinson–Gilford progeria syndrome and from xeroderma pigmentosum complementation groups A and C, were first restricted in number and then expanded in vitro. DNA was obtained from cells pre‐ and post‐expansion and sequenced at high depth (1656× mean coverage), over a cumulative 290 kb target region, including the exons of 44 aging‐related genes. Allele frequencies of 58 somatic mutations differed between the pre‐ and post‐cell culture expansion passages. Mathematical modeling revealed that the frequency change of three of the 58 mutations was unlikely to be explained by genetic drift alone, indicative of positive selection. Two of these three mutations, *CDKN2A* c.53C>T (T18M) and *ERCC8* c.*772T>A, were identified in cells from a patient with XPA. The allele frequency of the *CDKN2A* mutation increased from 0% to 55.3% with increasing cell culture passage. The third mutation, *BRCA2* c.6222C>T (H2074H), was identified in a sample from a healthy individual of advanced age. However, further validation of the three mutations suggests that other unmeasured variants probably provide the selective advantage in these cells. Our results reinforce the notions that somatic mutations occur during aging and that some are under positive selection, supporting the model of increased tissue heterogeneity with increased age.

## 1 INTRODUCTION

The aging process is characterized by a decline in tissue homeostasis, likely due in part to somatic mutations that arise and spread into future cellular generations as a consequence of mitosis (Failla, 1958; Szilard, 1959). Contrary to the assumption that all cells from a healthy individual have exactly the same genomic contents, each tissue of a given organism is actually a mosaic of cells with different mutational loads. Somatic mutation burden increases during postnatal tissue aging (Alexandrov, Nik‐Zainal, Wedge, Campbell, & Stratton, 2013; Blokzijl et al., 2016; Forsberg et al., 2012; Jacobs et al., 2012; Laurie et al., 2012; Lo Sardo et al., 2016; Martincorena et al., 2015; Milholland, Auton, Suh, & Vijg, 2015) as a result of normal and abnormal DNA replication, defective DNA repair, and exogenous and endogenous exposures that cause DNA damage. While somatic mutations are well studied in the context of cancer, the consequences of mosaicism for age‐related degeneration of generally healthy tissues and for etiology of other disease processes are less well understood (Jacobs et al., 2012; Laurie et al., 2012; Milholland et al., 2015; Vijg & Suh, 2013).

Efforts to characterize somatic mosaicism have been hindered by technical limitations of DNA sequencing assays to detect low‐frequency genetic variation within a tissue sample. In addition, low‐frequency somatic mutations have often been considered of limited biological significance, as long as the tissue seems to be functioning properly (Vijg & Suh, 2013). However, studies enabled by advances in deep sequencing have improved our understanding of the extent and effects of intra‐individual somatic genetic variation (Biesecker & Spinner, 2013; Forsberg et al., 2012; Jacobs et al., 2012; Jamuar et al., 2014; Laurie et al., 2012; O'Huallachain, Karczewski, Weissman, Urban, & Snyder, 2012; Roberts et al., 2015). Age‐related structural genetic changes and an increased fraction of cells with somatic mosaicism of large structural variants with increased age following serial sampling from the same individual argue for somatic variation as a contributor to tissue aging (Forsberg et al., 2012; Schick et al., 2013; Bonnefond et al., 2013; Machiela et al., 2015).

Hutchinson–Gilford progeria syndrome (HGPS, OMIM 176670) is a very rare disorder with several clinical features suggestive of premature aging. Xeroderma pigmentosum, complementation groups A and C (XPA and XPC, OMIM 278700 and 278720) are characterized by increased sensitivity to ultraviolet light and high incidence of skin cancer. Impaired DNA repair has been found in HGPS, XPA, and XPC and is believed to contribute to disease pathogenesis (Musich & Zou, 2009).

In this study, we investigate whether the frequency spectrum of genetic variation changes during in vitro aging, and whether that is affected by donor age, the presence of a germline mutation that promotes accelerated aging or by the presence of germline mutations that cause DNA repair deficiency. We used a deep sequencing approach to analyze intra‐individual genetic variation at the single nucleotide level in 44 age‐related candidate genes. Although deep whole‐genome sequencing provides high resolution and the ability to resolve rearrangements to the base pair, identifying lower level somatic changes from mosaic samples still remains a challenge. To circumvent this problem and to allow for an increased depth of analysis and sensitivity, we used target enrichment of genomic regions in combination with DNA extracted from both pre‐ and post‐cell culture expansion (early and late passage) primary cells from unaffected subjects of young and advanced age, and subjects with HGPS, XPA, and XPC. We identified a strongly enriched mutation in *CDKN2A* in an XPA patient and revealed a wide‐spread accumulation of low‐level somatic mutations during the in vitro cell aging process.

## 2 MATERIALS AND METHODS

### 2.1 Subjects

Primary dermal fibroblast cell cultures from individuals diagnosed with HGPS (ages 2–4 years, samples HGADFN188, HGADFN003, and HGADFN164), XPC (ages 3–5 years, samples GM16684 and GM16685), and XPA (age 8 years, sample GM02990) were obtained from the Coriell Cell Repository and the Progeria Research Foundation cell and tissue bank (Table 1). Primary dermal fibroblasts for the limited cell expansion also included samples from unaffected individuals of young (ages 11–13 years, samples GM01864, GM02036, and GM02037) and advanced age (ages 85–94 years, samples AG13077 and AG08433), obtained from the Coriell Cell Repository (Table 1).

**Table 1:** Early and late passage samples used in the study

State	Age (years)	Gender	Previously known mutation	Phenotype	Cell repository sample id
Young healthy	11	Male	–	–	GM01864
Young healthy	11	Female	–	–	GM02036
Young healthy	13	Male	–	–	GM02037
Advanced age healthy	85	Female	–	–	AG13077
Advanced age healthy	94	Male	–	–	AG08433
XPA	8	Female	*XPA* c.374delC	Onset of skin symptoms at one year of age; photophobia; microcephaly and mental deficiency probably from birth; 1 similarly affected sib; hypersensitive to UV cell killing and mutagenesis.	GM02990
XPC	5	Male	*XPC* c.669_670delAT	Developed skin cancer (basal cell carcinoma) at 2 years of age and died at 10 years of age; skin fibroblasts showed reductions in postultraviolet survival (11% of normal), unscheduled DNA synthesis (10% of normal), global genome DNA repair (15% of normal); and plasmid host cell reactivation (5% of normal).	GM16684 (sibling with GM16685)
XPC	3	Female	*XPC* c.669_670delAT	Developed skin cancer at 10 years of age; by age 24, donor subject had developed 10 skin neoplasms (five basal cell carcinomas, two squamous cell carcinomas, and three melanomas); skin fibroblasts showed reductions in postultraviolet survival (11% of normal), unscheduled DNA synthesis (10% of normal), global genome DNA repair (15% of normal); and plasmid host cell reactivation (5% of normal)	GM16685 (sibling with GM16684)
HGPS	2.25	Female	*LMNA* c.1824C>T; p.G608G	Classical form of HGPS	HGADFN188
HGPS	2	Male	*LMNA* c.1824C>T; p.G608G	Classical form of HGPS	HGADFN003
HGPS	4.67	Female	*LMNA* c.1824C>T; p.G608G	Classical form of HGPS	HGADFN164

DNA extracted from samples AG10579 and AG07091, obtained from the Coriell Cell Repository, was used in a frequency simulation doping experiment to assess the sensitivity of the low‐frequency variant detection method we used in the study.

### 2.2 Limited cell culture expansion and DNA preparation

**Figure 1:**
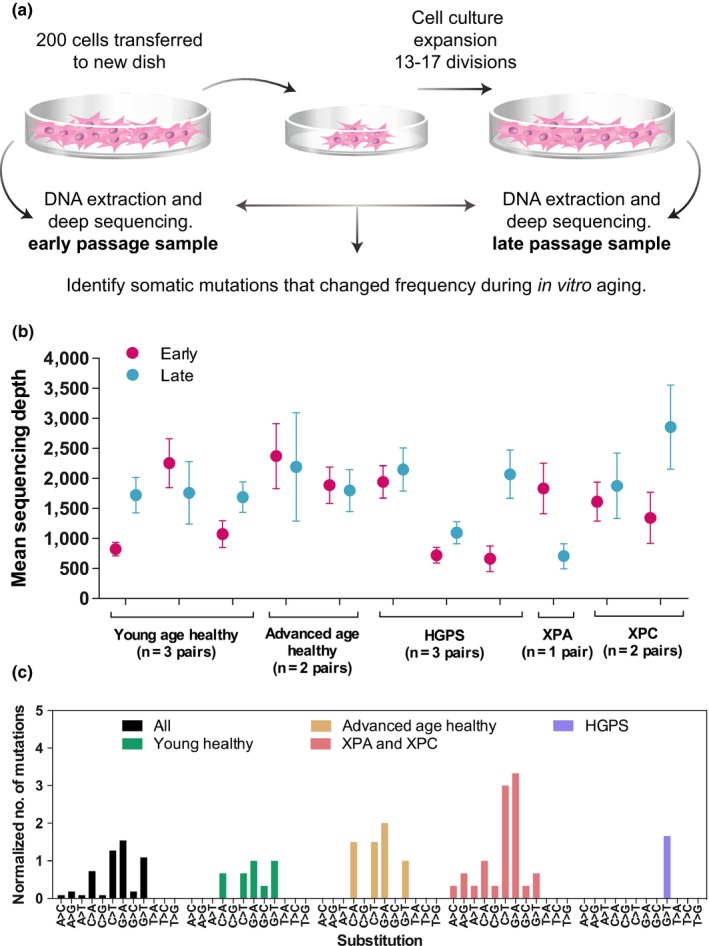
Detecting somatic mutations during in vitro aging. (a) Experimental setup for the collection of early and late passage cell culture expanded primary dermal fibroblasts. (b) The mean read depth across exons per sample. Early and late passage sample from the same cell culture were located next to each other. (c) Type of single nucleotide substitutions within the sample groups. The number of mutations was normalized to the number of cell pairs studied in individual sample groups

For the limited primary fibroblasts expansion, the cells were plated in individual wells of 200 cells per well and expanded in culture for 13–17 cell divisions (Figure 1a). The early (pre‐cell culture expansion) and late (post‐cell culture expansion) passage populations were defined as follows: early passage DNA was collected from cells at the same time point as when cells were harvested for plating for cell culture expansion. Late passage DNA was collected from the cells that had been expanded in culture for 13–17 cell divisions (Figure 1a). DNA was extracted using the Gentra Pure Gene Cell kit (Qiagen) according to the manufacturer's recommendations, but without the 65°C incubation step.

### 2.3 Target enrichment

NimbleGen SeqCap EZ Choice Library (Roche, Inc.) was used to capture a genomic target region of 290 kb. The target gene panel was made up of the coding region (exons and exon–intron boundaries) of 44 genes including genes involved in cell cycle regulation, DNA repair, telomere maintenance, and the nuclear lamina (Table 2; Table S1).

**Table 2:** Exons from the genes listed and the intron 11 of *LMNA* gene were targeted for sequencing using a hybridization selection array

Function	Gene
Telomere maintenance	*TERC, TERT*
DNA repair	*ATM, ATR, BRCA1, BRCA2, MSH2, XPA, RAD51, WRN, BLM, RECQL4, RECQL5, RECQL, ERCC8, ERCC6, ERCC3, ERCC2, GTF2H5*
Cell cycle regulation	*MYC, TP53, FOS, JUN, RB1, CDKN1A, CDKN2A*
Other, aging implicated	*IGF1, SOD1, ZMPSTE24, RCE1, LMNA, TP63, KL, SHC1, RPS6KB1, GHR, IGF1R, SIRT1, FOXO3, POLG, SIRT6, CCAR2, LMNB1, LMNB2*

### 2.4 Library preparation and sequencing of captured DNA

The library preparation was performed as per the manufacturer's protocol (NimbleGen SeqCap EZ Library SR User's Guide v3.0, Roche, Inc.). Briefly, genomic DNA (5–10 µg) of each cell culture was sonicated using a Covaris S2 instrument (Covaris, Inc.) to obtain fragments of approximately 200 bp. Then, sheared DNA samples were subjected to three enzymatic steps: end repair, A‐tailing, and ligation to Solexa paired‐end indexed adapters. Once the DNA libraries were indexed, they were PCR‐amplified for 8 cycles using the Tuft P1 and P2 primers, pooled, and in‐solution hybridized to a custom NimbleGen SeqCap EZ Choice Library (Roche, Inc.) of complementary oligonucleotide DNA baits. Following washing, the captured libraries were amplified by PCR using the Tuft P1 and P2 primers (12 or 24 cycles). Validation of enrichment of each sample for targeted and nontargeted genes was tested by quantitative PCR on captured libraries that were amplified by PCR (24 cycles). Samples that showed enrichments were amplified by 12 cycles, and pooled libraries (8 libraries/lane) were subjected to massively parallel sequencing using a 101‐bp paired‐end protocol on the HiSeq platform (Illumina, Inc.). The resulting fastQ files were analyzed as described below (Table S2).

### 2.5 Sequence analysis and somatic variant calling

We aligned the sequence reads of each library to the GRCh37 reference genome using novoalign v3 (Novocraft). We applied GATK (McKenna et al., 2010) base quality score recalibration and removed PCR duplicates. Subsequently, we used clipOverlap of bamUtil (http://genome.sph.umich.edu/wiki/BamUtil:_clipOverlap) to remove overlapping reads to reduce likelihood of double counting bases. Remaining aligned reads of the bam files were used in further analyses.

We took an empirical approach to discover somatic mutations that have gone through variant frequency change from one passage to the other. First, we used “lofreq somatic” (Wilm et al., 2012) to identify candidate somatic mutations that were detected in one passage, but not in the other of an individual (late passage compared to early passage or vice versa) above the level of sequencing error as determined by the algorithm. Briefly, this approach involves two steps to generate an initial list of mutations: (a) calling variant alleles at each site in a passage with a *p*‐value < .01 based on Poisson binomial distribution of phred‐scaled quality scores of the variant alleles; (b) filtering out any sites where the same variant allele is present in both passages of an individual. Next, we manually examined mutations identified in both passages of any individual to look for possible selection, seeking to identify sites that had a variant allele frequency difference between passages >3 standard deviations from the mean across all sites. Only one variant was identified that met this criterion and that was added to the candidate mutation list. We refer to the final list of somatic mutations as “SM‐Vs” to distinguish them from somatic mutations present at similar rates throughout two passages. Only those SM‐Vs identified at genomic sites with expected homozygous genotypes (see below) were retained for further analysis. SAMtools (Li et al., 2009) was used to obtain variant allele counts of the identified SM‐Vs in each passage library. Reads with mapping quality score <30 and bases with quality score <30 were excluded from any coverage counts. Finally, each SM‐V was manually checked in the sequence reads using “samtools tview” to ensure no excess of mutations were observed in a 50 bp neighborhood. ANOVAR (Wang, Li, & Hakonarson, 2010) was used to annotate the functional significance of the variants based on the hg19 UCSC known gene database.

The Bayesian based most probable genotype calling algorithm implemented in mpg (Teer et al., 2010) was used to call diploid genotypes of each library from the sequence reads with mapping score ≥30 and bases with quality score of ≥30. Genotypes with mpg quality score ≥10 were used for further analyses. Genotypes of early and late passage libraries of all individuals were compared pairwise to ensure no sample swap or DNA mishandling occurred.

### 2.6 Assessing limit of detection for mosaicism

To evaluate the sensitivity of our approach in calling low‐level somatic mutations, we generated two mosaic control libraries by diluting DNA from one individual (host DNA, AG10579) with DNA from a second subject (doped‐in DNA, AG07091; Table S3). These two control libraries were prepared and sequenced to a comparable depth to the other experimental samples described above. The resulting libraries of mixed DNAs are expected to generate variant alleles of the doped‐in DNA at frequencies of ≥0.1% and ≥0.2% at genomic sites where the two DNAs differ. Genomic sites with variant alleles (alternate allele frequency ≥1) were identified using “samtools mpileup” in each of the mosaic libraries. The same read and base quality filtering was applied to the sequence reads in variant calling as previously described. Only the sites where genotypes of the host DNA are homozygous were considered for subsequent evaluation (see below for calling diploid genotypes of the two DNAs involved).

Since ≥99.6% of input DNA of the mosaic libraries came from the host DNA, diploid genotypes of the host DNA were determined from the sequence reads of the mosaic DNAs using mpg as described above. The doped‐in DNA sample was genotyped at the Genetic Resources Core Facility (GRCF) of the Johns Hopkins Institute of Genetic Medicine on the HumanOmniExpress v1_H BeadChip array (Illumina). We mapped the Illumina array probe sequences to the GRCh37 genome assembly using BWA (Li & Durbin, 2009). We excluded SNPs with probe alignment problems or with known variants within 7 bp of the 3′ end of probes.

Based on the mutations identified in the mosaic DNAs and diploid genotypes of the two samples involved, we calculated sensitivity (true‐positive rates, proportion of sites with variant alleles out of the expected ones) and specificity (true‐negative rates, proportion of sites with no mutation out of the sites where no variant allele is expected).

### 2.7 Mathematical modeling of genetic drift

In order to assess whether the observed change of variant frequency between the early and late passage cells per individual/patient at a given site was due to cell growth expected under the random process of genetic drift, we carried out an in silico experimental process of cell growth. We calculated the probability, given the early passage variant allele count, of observing a late passage variant allele count at least as often as that observed in the data under the null model of basic genetic drift. To calculate this probability, we constructed a mathematical model of a population with a single constant size bottleneck, created by binomially sampling 200 cells, and genetic drift, using a modified Moran model of population growth, to build the late passage cells (see Appendix S1). This corresponds to a discrete Markov chain with two transition matrices for the bottleneck and subsequent growth. In addition, we incorporated terms for the sampling and sequencing error expected in the experiment. Constructing a closed‐form equation, we calculated a *p*‐value: the probability of observing the change in allele frequency under the null hypothesis of genetic drift alone. Because a total of 290 kb genomic region was chosen for the targeted sequencing and further assessed for identification of possible somatic mutations, we adjusted for multiple testing by comparing each *p*‐value to a Bonferroni‐corrected alpha (*α* = 0.05/2.9 × 10^5^ = 1.7 × 10^−7^). Custom codes used in the modeling are available at: https://github.com/rsrothwe/Aging.

When this probability is significantly small, indicating sites that accumulate or deplete significant number of variant alleles, we incorporated and estimated a selection coefficient. We defined the selection coefficient, *s*, such that cells with 1 copy of the allele should produce a factor (1 + *s*) more offspring. We incorporated this factor into the modified Moran model of population growth, constructing a likelihood equation for each possible value of *s*. To estimate *s*, we applied a grid search for *s*=[−1.0, 1.0] to maximize this likelihood and calculate a 95% confidence interval based on the log‐likelihood ratio test using a chi‐square statistic with 1 degree of freedom (see Appendix S1).

### 2.8 Validation of somatic mutations

Digital droplet PCR (ddPCR, Bio‐Rad) was used to validate rare somatic mutations identified during deep sequencing (Hindson et al., 2011). Primers and probes for ddPCR were designed by the technical support at Bio‐Rad (Table S4). ddPCR was performed on the same batch of genomic DNA that was used for the library preparation using 900 nM forward and reverse primers, and 250 nM mutant and wild‐type genomic probes. DNA fragmentation by restriction enzyme digest in the ddPCR was achieved by the addition of HindIII (5 U/reaction; New England Biolabs) and incubation at RT for 5 min. The PCR was performed with annealing/extension temperatures of 52.4–56.2°C for 40 cycles. The QX200 Droplet Digital PCR Systems (Bio‐Rad) were used for droplet generation and analysis.

For two mutations, we were unable to use functional ddPCR assays due to the nucleotide composition of the surrounding sequence (Table S5). The *CDKN2A* c.53C>T mutation was thus validated using Sanger sequencing. PCR primers used to amplify the region containing the mutation included M13 tags and were as followed: forward 5′‐GTAAAACGACGGCCAGTCCGTAACTATTCGGTGCGTTG‐3′ and reverse 5′‐GGAAACAGCTATGACCATGTAATAGCACCTCCTCCGAGC‐3′. *TERT* c.121C>A mutation was validated by restriction fragment length polymorphism analysis (PCR‐RFLP) using BsrBI restriction enzyme (5 U/reaction; New England Biolabs). The region containing the mutation was amplified prior to digestion using the following primers: forward 5′‐GAGCCACCAGCACAAAGAG‐3′ and reverse 5′‐GCGCGAGTTTCAGGCA‐3′.

### 2.9 RNA extraction and gene expression analysis

Frozen back‐up aliquots of early and late passage cell cultures were thawed, expanded, and passaged one passage. Total RNA was extracted using TRIzol reagent (Invitrogen) according to the manufacturer's recommendations. cDNA was synthesized from 750 ng of total RNA using SuperScript First‐Strand Synthesis System for RT–PCR (Invitrogen) according to the manufacturer's recommendations. Absolute quantification of *CDKN2A* transcript levels was performed using ddPCR with two sets of previously published primers: forward 5′‐CCCAACGCACCGAATAGTTAC‐3′ (primer 3), reverse 5′‐CACGGGTCGGGTGAGAGT‐3′ (primer 4) (Shibata et al., 2007); and forward 5′‐CAACGCACCGAATAGTTACGGTC‐3′ (primer 2), reverse 5′‐TCTATGCGGGCATGGTTACTG‐3′ (primer 5) (Bostrom, 2001). *GAPDH* was used as a housekeeping gene with previously published primers: forward 5′‐GAGCGAGATCCCTCCAAAAT‐3′ and reverse 5′‐CATCACGCCACAGTTTCC‐3′ (Revêchon et al., 2017). ddPCR was performed according to the manufacturer's recommendations on 4.75 ng (for *CDKN2A*) or 0.475 ng (for *GAPDH*) of cDNA, respectively, with 100 nM forward and reverse primers and annealing/extension temperatures of 62°C or 60°C, respectively, for 40 cycles. To distinguish between the expression of the mutated and wild‐type *CDKN2A* alleles, we performed restriction fragment length polymorphism analysis. 40 ng of cDNA was first amplified using the following primers: forward 5′‐TAATAGCACCTCCTCCGAGC‐3′ (primer 1) and reverse primer 5. After clean‐up with QIAquick PCR purification kit (Qiagen) according to the manufacturer's recommendations, 25% of the PCR product was digested using EagI restriction enzyme (10 U/reaction; New England Biolabs) and visualized on 2% agarose gel.

## 3 RESULTS AND DISCUSSION

### 3.1 Deep sequencing

In this study, we developed a model to study the extent to which somatic mutations occur in exons of a selection of genes across the genome during in vitro aging of human skin fibroblasts (Table 2, Table S1). Primary dermal fibroblasts from two different age‐groups of healthy individuals (11–13 and 85–94 years of age) were included (Table 1). In addition, patient samples from genetic disorders with accelerated aging and/or genomic instability, including HGPS, XPC, and XPA, were included, as positive controls, anticipating the possibility of increased somatic mutation events in these patients (Table 1; Lodato et al., 2018; Mukherjee & Costello, 1998) To limit the initial cellular somatic genotypes and to explore the possibility of selection, we introduced an artificial bottleneck: two hundred early passage cells were sub‐cultured and expanded in vitro for 13–17 cell divisions (Figure 1a). In initial attempts, smaller starting populations of cells were tested, but impaired growth of patient cells showed that 200 cells were the minimum number of cells needed to be able to obtain sufficient amounts of late passage cells for all the samples.

Target gene capture and high‐throughput sequencing resulted in an average of 34.33 million mapped reads per sample (Table S2). After removal of PCR duplicates and reads derived from outside our targeted region, there were 6.6 million mean unique reads within the region of interest across all the samples. The mean read depth across all samples was 1656× (in a range of 661–2856×; Figure 1b).

### 3.2 Assessment of limits of mutation detection

In our two mosaic DNA libraries with 0.2% or 0.4% of doped‐in second DNAs, we obtained sensitivity ≥74% in detecting variant alleles (Table S3) and specificity ≥87%, indicating that we have sufficient power to detect likely true variants. All sites with false‐positive calls in these two libraries have mutation allele count ≤2. This shows that a detected mutation could be potentially false when very few copies of variant alleles are present. However, all SM‐Vs detected to differ in variant frequency between two passages in the experimental samples for subsequent analyses have ≥8 copies and variant frequency >0.48% in at least one of the stages.

### 3.3 Detection of variable frequency somatic mutations (SM‐Vs)

In the two passages of 11 subjects, “lofreq somatic” identified 57 somatic mutations that were absent in one passage from an individual, but present in the other passage. In addition, one mutation that was present in both passages of an advanced age healthy subject (AG13077), but had a significant difference in allele frequency, was retained by our manual check (*BRCA2*, chr13:32914714, c.6222C>T, H2074H). These 58 SM‐Vs are summarized in Table S6. Further evaluation of the relevant sequence reads did not find anomalies in the reads and bases involving these mutations.

Eleven SM‐Vs with varying allele frequencies (ranging from 0.66% to 55.3%) were selected among the variants that differed in allele frequency between early and late passage for validation by ddPCR (Table S5). DNA used for the library preparation was also used for the validation to test for artifactual variants included during the library preparation. Eight of the mutations were validated by ddPCR. The fractional abundances obtained were in a similar range to that seen with deep sequencing (Table S5). One of the mutations, *ERCC3* c.505G>T, p.V169F, was not detected by ddPCR (Table S5). Two of the mutations failed ddPCR due to technical limitations with the assays and were instead validated by Sanger sequencing and RFLP analysis. The results from the Sanger sequencing and RFLP analysis confirmed the presence of the mutations (Table S5). Hence, all but one of eleven selected mutations was validated by an alternative method.

The majority of the 58 SM‐Vs had an unknown impact (34/58); those were located in 3′ or 5′UTR (22/34), intergenic (2/34), or intron (10/34). The remaining 23 mutations, in exons, were missense (15/58), synonymous (6/58), or splice consensus mutations (2/58). There were also dinucleotides (12/58) or splice consensus mutations in intronic regions (1/58) (Table S6). The majority of the mutations were in the samples from the XP patients (30/58, including XPA 17/58 and XPC 13/58), while the number of variants were similar for the other groups: HGPS (5/58), young healthy (11/58), and advanced age healthy (12/58). The predominance of mutations in the XP patient cell lines was expected, due to their inherent genomic instability. These findings are consistent with a recent study showing 2.5‐fold higher numbers of somatic mutations in XP patient neurons compared to expected age‐adjusted controls (Lodato et al., 2018). On the other hand, the relative distribution of the remaining mutations suggested that, at least in this sample population, using this in vitro assay, and for the genomic regions studied here, that there was no evidence to support the conclusion that the HGPS patients had increased genetic instability compared to healthy age‐matched subjects, or that cells from advanced aged healthy subjects were less genetically stable compared to young healthy subjects during in vitro aging. Several studies have shown that somatic mutations accumulate with aging in vivo with an estimated constant rate of approximately 13–40 mutations per cell per year, regardless of age (Blokzijl et al., 2016; Franco et al., 2018; Lodato et al., 2018; Osorio et al., 2018). But while cells from aged individuals may have accumulated more mutations, there is conflicting evidence whether the rate of acquiring new mutations goes up or not (Milholland et al., 2015; Podolskiy, Lobanov, Kryukov, & Gladyshev, 2016). It is entirely possible that in our study, the number of cell divisions that occurred during in vitro aging was too low to induce enough somatic mutations to reliably distinguish between HGPS, young age healthy, and advanced age healthy samples, which are expected to have small differences in genomic integrity. In addition, our study only targeted a small region of the genome.

The number of variants in each sample group was low, and examination of the types of substitutions within each sample group did not identify specific patterns (Figure 1c). Of the 23 mutations in exons, there were 15 transitions and 8 transversions. Interestingly, the XP patients had mostly transitions (92% of the exonic mutations) while the young and advanced aged healthy had lower frequencies of exonic transitions (25%) or the same frequency of transitions and transversions, respectively (Table S6).

The dinucleotide mutations were equally distributed between CC>TT (3/6) and GG>AA (3/6). The CC>TT mutations were only found in XP cells, which is in agreement with previous data that these tandem mutations are induced by UV radiation exposure or oxidative stress, and occur at a higher frequency in XP patients that have deficient nucleotide excision repair (Marteijn, Lans, Vermeulen, & Hoeijmakers, 2014). Five of the mutations had previously been found in different types of tumors, as reported in the COSMIC database (Table S6).

### 3.4 Modeling of bottlenecks and genetic drift

For each of the 58 SM‐Vs identified by lofreq, we assessed the evidence against a null model of genetic drift. Twenty of the mutations showed nominally significant departure from this null hypothesis, but only three of them showed significant evidence after correcting for multiple testing (*p*‐value < 1.7 × 10^–7^; Table 3, Table S6). All three of these significant mutations increased in frequency from early to late passage. Two of the three mutations were not detected in the initial sequenced sample, thus likely having a very low initial allele frequency. The most significant *p*‐value was for mutation chr9:21974774:A (*CDKN2A* c.53C>T), with a variant allele frequency changes from 0% to 55.3% (Table S6). While the remaining 55 SM‐Vs did not pass a Bonferroni‐corrected significance level, 36 SM‐Vs had decreased variant allele frequency during cell growth, and the remaining ones had higher frequency in the late passage cells.

**Table 3:** Somatic mutations (SM‐Vs) with significant change of variant allele frequency between two stages

Mutation	Variant ID number	Sample id	Phenotype	Initial population sample			Final population sample			*p*‐Value	MLE of selection coefficient	95% confidence interval
				Minor allele count	Total count	Minor allele frequency (%)	Minor allele count	Total count	Minor allele frequency (%)			
*CDKN2A* c.53C>T	chr9:21974774:A	GM02990	XPA	0	725	0	156	282	55.32	9.82E−39	0.5	0.4, 0.8
*ERCC8* c.*772T>A	chr5:60169670:T	GM02990	XPA	0	2,168	0	122	705	17.3	3.02E−14	0.3	0.2, 0.6
*BRCA2* c.6222C>T	chr13:32914714:T	AG13077	Advanced age healthy	42	3,122	1.35	642	2,996	21.43	3.87E−13	0.25	0.2, 0.4

For the three SM‐Vs, we estimated the maximum likelihood of the selection coefficient. All three of the variants have estimates of strong positive selection (*s* > 0.25) (Table 3). The variant with highest significance value has the strongest selection coefficient estimate of 0.5. For these variants, we also calculated the 95% confidence intervals of the selection coefficient.

### 3.5 Functional significance of detected mutations

**Figure 2:**
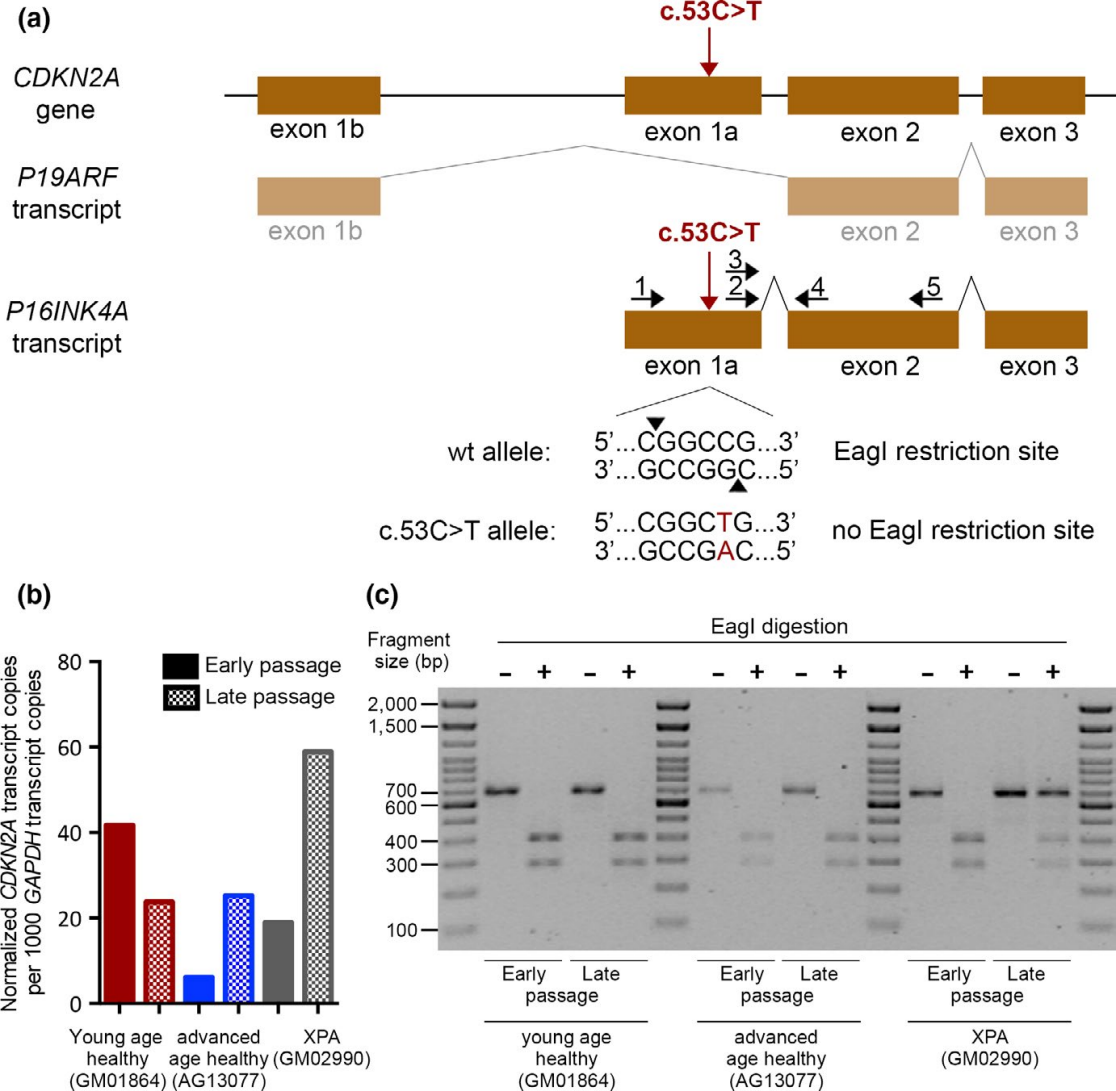
*CDKN2A* gene and its expression in the presence or absence of the c.53C>T mutation. (a) Schematic representation of the genomic structure and transcripts of the *CDKN2A* gene locus with c.53C>T mutation and primer locations. The exons are shown as rectangles and primers as arrows. Primers 1 and 5 were used for RFLP analysis (see figure c), primers 3 and 4 were used for ddPCR analysis (see figure b), and primers 2 and 5 were used for additional ddPCR analysis (see Figure S1). An EagI restriction site is present in a wild‐type carrier, but absent in the individual with the c.53C>T mutation. The size of the locus, transcripts, exons, and introns is not shown to scale. Of note, the size of the intron between exon 1a and exon 2 is approximately 3.5 kb. (b) Comparison of *CDKN2A* transcript copies after normalization to *GAPDH* in early and late passage primary fibroblasts from young and advanced age healthy subjects without the c.53C>T mutation and in early and late passage primary fibroblasts from the XPA patient where the c.53C>T mutation is present at an allele frequency of 55.3% in the late passage. Primers 3 and 4 were used for this ddPCR analysis. (c) RFLP analysis of *CDKN2A* transcript in early and late passage primary fibroblasts from young age healthy and advanced age healthy subjects without the c.53C>T mutation and in early and late passage primary fibroblasts from XPA patient where the c.53C>T mutation is present at an allele frequency of 55.3% in the late passage only. Primers 1 and 5 were first used to amplify the cDNA followed by digestion with restriction enzyme EagI (indicated by +). Part of the amplified sample was used as an undigested control (indicated by −). Undigested PCR product is 658 bp in size, while digested fragments are 274 bp and 384 bp in size

Following mathematical modeling, three mutations remained that had allele frequencies that differed and could not be explained solely by genetic drift (Table 3). The most striking differences in allele frequencies between early and late passage (0% and 55.3%) were observed for the *CDKN2A* c.53C>T, p.T18M mutation during in vitro aging of dermal fibroblasts from an XPA patient (Tables S5 and S6). The mutation is a C>T substitution in a CpG dinucleotide, located in a CpG island (ENCODE Consortium, 2012, https://genome.ucsc.edu). CpG islands are considered mutational hotspots (15 times higher mutation rate than the genome‐wide average) resulting from spontaneous deamination of 5‐mC that resolves as a C>T transition mutation (Kim, Elango, Warden, Vigoda, & Yi, 2006; Kondrashov, 2003). This mutation is located in the part of the *CDKN2A* gene that is not highly mutated in cancer (https://cancer.sanger.ac.uk/cosmic), although it has been identified in one liver carcinoma case and suggested it might lead to *CDKN2A* inactivation (Anzola, Cuevas, Lopez‐Martinez, Martinez de Pancorbo, & Burgos, 2004). Missense mutation prediction algorithms (Polyphen‐2, http://genetics.bwh.harvard.edu/pph2/; SIFT, http://sift.bii.a-star.edu.sg), however, point toward a minimal effect on protein function. While these predictions do not definitively exclude the possibility of the variant affecting protein function, that seems unlikely. Further bioinformatic analysis of the genetic region indicates that the mutation is located in a DNase hypersensitive site (DHS) (ENCODE), which suggests the presence of a regulatory element. The mutation also lies within the binding site of several transcription factors, including *EZH2. EZH2* is a known inhibitor of the INK4A‐ARF pathway (it has the highest rank for this particular position), and its binding has also been confirmed in several fibroblast cell lines (Bracken et al., 2007; ENCODE Consortium, 2012). So even though the *CDKN2A* c.53C>T, p.T18M mutation changes the amino acid sequence, we considered that its functional consequence might also relate to an effect on transcription. With that in mind, we selectively evaluated *P16INK4A* transcript expression in early and late passage samples from this XPA patient and compared it to *P16INK4A* expression in one young age healthy and one advanced age healthy individual (Figure 2a,b and Figure S1). We performed the analysis with two different primer sets and both showed that expression of *P16INK4A* modestly increased in the late passage samples when compared to their early passage counterparts from advanced age healthy individual and XPA patient. This indicates that the c.53C>T mutation did not reduce and might have even contributed to the increased *P16INK4A* expression. Furthermore, looking at the expression of mutant and normal *CDKN2A* alleles separately, it was clear that the mutant allele remained strongly expressed. Similar expression of both alleles indicates that mutation did not have a significant cis‐acting effect on gene transcription (Figure 2a,c). We conclude that it is unlikely that the *CDKN2A* c.53C>T, p.T18M mutation contributed to the observed clonal expansion.

The *CDKN2A* mutation was frequently coincident in the same patient (GM02990) with another mutation, *ERCC8* c.*772T>A, located in the 3′UTR. The *ERCC8* mutation differed in allele frequencies between early and late passage (0% and 15.7%, respectively), obtained from the fractional abundances during ddPCR validation (Table S5). Analysis of the possible functional impact of the *ERCC8* mutation revealed that it is located within seed regions of two micro‐RNAs, miR‐376c and miR‐4803 (ENCODE, TargetScan http://www.targetscan.org/vert_72/; miRWALK (http://zmf.umm.uni-heidelberg.de/apps/zmf/mirwalk2/; miRANDA, http://www.mirdb.org), and thus could be an important target of posttranscriptional regulation of the gene during in vitro aging.

**Figure 3:**
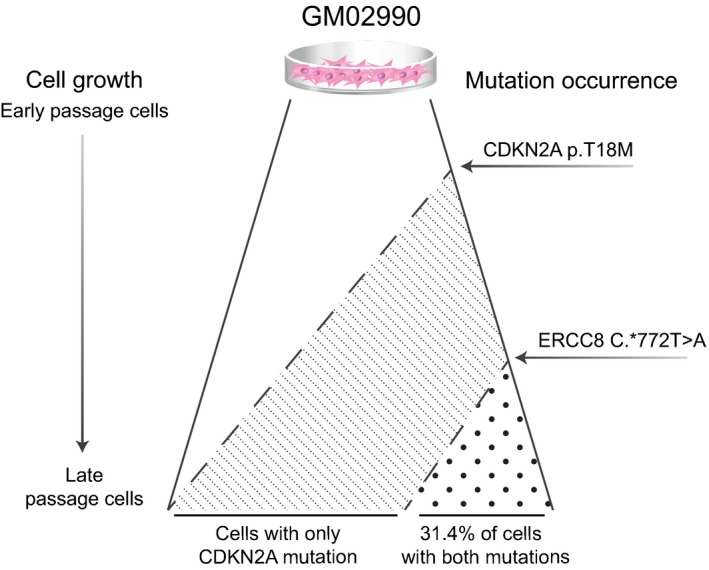
A model for the reoccurrence of somatic mutations in the same cell from an XPA patient. Both the *CDKN2A* c.53C>T, p.T18M and *ERCC8* c.*772T>A mutations had changes in allele frequencies (0%–55.3% and 0%–15.7%, respectively) that suggest they occurred in a cell with a strong competitive advantage. Based on the allele frequencies, we propose that *CDKN2A* mutation occurred at the very beginning of the in vitro aging while the *ERCC8* mutation occurred later on during cell culturing, in a cell that already encountered another unknown driver event

Neither an examination of sequence reads in the target region of *CDKN2A* mutation nor a comparison of the read coverage between different passages of the same individual and with libraries from another individual support a hypothesis that the absence of the mutation at the early passage was caused by a deletion. Instead, the observation of a ~50% mutation rate in the late stage cells suggests that this *CDKN2A* mutation has essentially gone to fixation, and virtually all cells are now heterozygous. While *CDKN2A* would be a plausible driver of clonal expansion, due to its role in the cell cycle and its involvement in a variety of human cancers, there was no evidence of reduced expression despite the presence of the mutation (Anzola et al., 2004). We therefore hypothesize that the *CDKN2A* and the *ERCC8* mutations occurred as independent spontaneous events, the former mutation at the very beginning of the in vitro cell culture experiment and latter later on (Figure 3), and have been carried along as innocent bystanders by a cell clone that was being driven by another unmeasured driver event. For example, mutations in *KRAS* and *BRAF* genes often co‐occur within cells and drive expansion of *CDKN2A* mutations in pancreatic cancer and cutaneous melanoma, respectively (Akbani et al., 2015; Bruno, 2018; Hayashi et al., 2017; Yachida et al., 2012).

*BRCA2* c.6222C>T, p.H2074H was the third SM‐V that was indicated by its change in allele frequencies to provide a selective advantage over the growth (1.59% and 21.3%, respectively, obtained from the fractional abundances during ddPCR validation, Table S5). This mutation was found in a sample from a healthy subject of advanced age (85 years old). While this is a synonymous mutation and might therefore be expected to be neutral, analyzing the functional significance of the *BRCA2* mutation showed that it could potentially cause aberrant splicing of the C‐terminal region (Human Splicing Finder, Alamut splicing tools). That region is essential for *BRCA2* function and its interaction with *RAD51* (O'Donovan & Livingston, 2010). Alternatively, it is possible that this mutation is traveling on a *BRCA2* haplotype that carries other unmeasured variants providing selective advantage.

## 4 CONCLUSIONS

In this work, we designed a method based on deep targeted sequencing to detect rare variants that occur during in vitro aging. An important part of the work was to develop a population genetics model for tracking somatic variants in cell culture. We used this to estimate the statistical significance of somatic mutations with major differences in allele frequency between early and late cell passages. Not unexpectedly, modeling indicated that most mutations (55/58 SM‐Vs) are indistinguishable from random events based on genetic drift; nevertheless, we identified three mutations with significant allele frequency changes that might represent a signature of positive selection. It is not surprising that two out of these three de novo mutations were identified in primary fibroblasts with inherited DNA repair deficiency. The high allele frequencies in these lines represent a signature of a clonal event, but in each instance, it seems most likely that another mutational event, outside the target area of our genome sequencing, represents the actual driver. Future studies are needed to further define the significance of somatic mutations in cells of various lineages for a wide range of disorders and aging. This work was limited to a selected set of genes associated with aging and to in vitro induced aging that spanned relatively few cell divisions. Subsequent studies will need to take advantage of the increased availability and low cost of DNA sequencing to look at these issues across the entire genome of multiple different cell types.

## CONFLICT OF INTEREST

Authors declare that there is no conflict of interest.

## Data Availability

BAM files of DNA sequence reads for all samples used in the paper have been deposited in NCBI dbGaP database with the accession code phs001867.v1.p1 and are available via the repository's access request procedures.
