# Supplementary figures and images for: Analysis of somatic mutations identifies signs of selection during in vitro aging of primary dermal fibroblasts
Source: Aging Cell. 2019 Aug 5;18(6):e13010. doi: 10.1111/acel.13010 (PMC6826141; doi:10.1111/acel.13010)

normalized *CDKN2A* transcript copies  
per 1000 *GAPDH* transcript copies

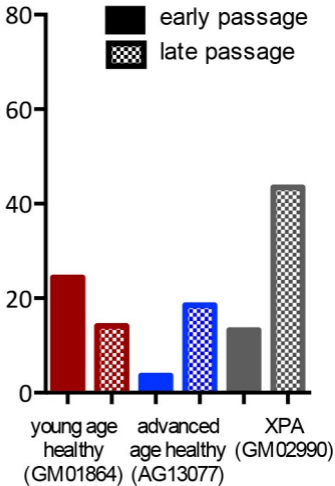

Supplement: Supplementary file 1 [file ACEL-18-e13010-s001.pdf]
