# Supplementary material for: Analysis of somatic mutations identifies signs of selection during in vitro aging of primary dermal fibroblasts
Source: Aging Cell. 2019 Aug 5;18(6):e13010. doi: 10.1111/acel.13010 (PMC6826141; doi:10.1111/acel.13010)
Supplement: Supplementary file 8 [file ACEL-18-e13010-s008.docx]

Supplementary Materials for

**Analysis of somatic mutations identifies signs of selection during in vitro aging of primary dermal fibroblasts**

Narisu Narisu^1^, Rebecca Rothwell^2^, Peter Vrtačnik^3^, Sofia Rodriguez^3^, John Didion^1^, Sebastian Zöllner^2,4^, Michael R. Erdos^1^, Francis S. Collins^1*^, Maria Eriksson^1,3^

**Mathematical Modeling of Population Bottlenecks and Genetic Drift**

We first describe the basic model of a single bottleneck of constant size $n_{b}$ with subsequent genetic drift (population growth) and no selection. The observed data set includes the following statistics for each variant: in the initial population (“early stage”) we observe a sample of size $n_{I}$ with observed minor allele count $k_{I}^{obs} (0\leq k_{I}^{obs}\leq n_{I})$; in the final population (“late stage”) we observe a sample of size $n_{F}$, with observed minor allele count, $k_{F}^{obs}(0\leq k_{F}^{obs}\leq n_{F})$,. We aim to directly formulate the probability, $P\left( k_{F}^{obs} | k_{I}^{obs}, n_{I}, n_{F}, n_{b} \right)$, the probability of observing the final minor allele count, given the bottleneck size and initial population statistics.

There are four primary components to this model: genotyping, sampling, population bottleneck, and genetic drift. In Figure A.1, we show a schematic diagram of how these components fit together. The observed initial minor allele count in the sample is obtained from the true minor allele count in the sample by genotyping, with some potential for error. This true minor allele count arises through sampling from the initial population, creating sampling error. This initial population undergoes the bottleneck, followed by subsequent genetic drift or population growth to obtain the final population. Similar to the initial population, this final population is also sampled and then genotyped, with uncertainty incurred at each step. Finally, this results in the final observed minor allele count. We will discuss each of these components separately, beginning with the primary population genetic processes, the bottleneck and genetic drift, and returning to the full probabilistic model at the end of this section. We include Table A. **1** as a reference for the symbols used to construct this model.

$k_{I}^{obs}$: Observed Minor Allele Count in **Sample** of Initial Population

$k_{F}^{obs}$: Observed Minor Allele Count in **Sample** of Final Population

$k_{I}$: True Minor Allele Count in **Sample** of Initial Population

$k_{F}$: True Minor Allele Count in **Sample** of Final Population

$K_{0}$: Minor Allele Count in **Initial Population**

$K_{F}$: Minor Allele Count in **Final Population**

Bottleneck: $n_{b}$ transmitted, $k_{b}$ with minor allele

Genetic drift (population growth) to full size final population

*Genotyping*

*error*

*Sampling*

*error*

*Sampling*

*error*

*Genotyping*

*error*

**Figure A.1 Schematic Diagram of Each Component of the Mathematical** Model We model genotyping, sampling, the population bottleneck, and genetic drift. The black boxes indicate the observed data: the initial population observed minor allele count and the final population observed minor allele count. The grey boxes highlight the primary population genetics components of the model: the bottleneck and subsequent genetic drift.

**Table A. 1 Symbols for Probabilistic Model**

| Symbol | Known or Unknown? | Meaning |
| --- | --- | --- |
| $k_{I}^{obs}$ | Known | Observed minor allele count in the sample of the initial population |
| $n_{I}$ | Known | Initial sample size |
| $k_{I}$ | Unknown | True minor allele count in the sample of the initial population |
| $k_{F}^{obs}$ | Known | Observed minor allele count in the sample of the final population |
| $n_{F}$ | Known | Final sample size |
| $k_{F}$ | Unknown | True minor allele count in the sample of the final population |
| $k_{b}$ | Unknown | Minor allele count in the post-bottleneck population |
| $n_{b}$ | Known | Post-bottleneck population size |
| $K_{F} (=k_{b}+z)$ | Unknown | True minor allele count in the full final population |
| $N_{F} (=n_{b}+j)$ | Known | Final Population Size |
| $j$ | Known | Number of steps during genetic drift |
| $z$ | Unknown | Additional minor alleles gained during genetic drift |
| $\varepsilon$ | Known | Position-specific error rate |
| $p_{F} \left( =\frac{K_{F}}{N_{F}} \right)$ | Unknown | Minor allele frequency in final population |
| $p_{0}$ | Unknown | Minor allele frequency in initial population |

*Population Bottleneck*

The bottleneck step is a random sample from the full size initial population. Let $N_{0}$ be the number of individuals in the full-size initial population and $K_{0}$ ($0\leq K_{0}\leq N_{0})$ of these individuals carry a minor allele. From this pool of individuals, we assume an unordered draw without replacement for $n_{b}$ individuals, $k_{b}$ (${0\leq k_{b}\leq n}_{b})$ of which carry the minor allele. In this case, $n_{b}=200$randomly chosen cells from the early stage cell population. Therefore, the number of individuals carrying a minor allele in the post-bottleneck population follows a hypergeometric distribution, $k_{b}\sim Hypergeometric\left( n_{b}, K_{0}, N_{0} \right)$. Where $N_{0}$ is much larger than $n_{b}$, this hypergeometric distribution converges in distribution to the simpler binomial distribution ($k_{b}\sim Binomial(n_{b},p_{0}=\frac{K_{0}}{N_{0}})$)^1^ as in (1). As in this application $n_{b}<<N_{0}$, we use this binomial distribution to model this bottleneck process, creating a transition matrix to all possible values of $k_{b}$ given $n_{b}, K_{0}, N_{0}$, with transition probabilities $P\left( k_{b} \right|n_{b}, p_{0}=K_{0}/N_{0})$.

|  | $P\left( k_{b} \vert p_{0}, n_{b} \right)=\left( \begin{matrix} n_{b} \\ k_{b} \end{matrix} \right)p_{0}^{k_{b}}\left( 1-p_{0} \right)^{n_{b}-k_{b}}$ | (1) |
| --- | --- | --- |

*Genetic Drift*

To model the subsequent genetic drift during the replication or population growth process, we build on the basic Moran model while incorporating a growing population size. Under the original Moran model, at each step, one individual dies and is replaced by the copy of another individual in the population^2^, maintaining a constant population size. In our modified Moran model, at each step, one individual is chosen randomly from the current population to be replicated and added to the current generation. This means there are no deaths, causing the population to grow by one individual in each step. Therefore, given a previous population size of $n_{b}$ with $k_{b}$ individuals carrying the minor allele, the current population of $n_{b}+1$ individuals can have $k_{1}$ individuals carrying the minor allele, where $k_{1}=k_{b}$ or $k_{1}=k_{b}+1$. This gives the following simple Bernoulli distribution for the number of minor alleles in the population after one step ($k_{1}$):

|  | $P\left( k_{1} \right\vert n_{b}, k_{b})=\left\{ \begin{matrix} \frac{k_{b}}{n_{b}} & k_{1}=k_{b}+1 \\ \frac{n_{b}-k_{b}}{n_{b}} & k_{1}=k_{b} \\ 0 & otherwise \end{matrix} \right\}$ | (2) |
| --- | --- | --- |

Similarly, in the next step, the proportions of minor and major alleles in the population are updated and the sampling repeats. Therefore, the number of minor alleles, $k_{2}$, in the population after two steps (population size now $n_{b}+2)$ is:

|  | $P\left( k_{2} \right\vert n_{b}, k_{b})= \left\{ \begin{matrix} \frac{n_{b}-k_{b}}{n_{b}}\left( \frac{n_{b}-k_{b}+1}{n_{b}+1} \right) & if k_{2}=k_{b} \\ 2\frac{k_{b}\left( n_{b}-k_{b} \right)}{n_{b}\left( n_{b}+1 \right)} & if k_{2}=k_{b}+1 \\ \frac{k_{b}}{n_{b}}\left( \frac{k_{b}+1}{n_{b}+1} \right) & if k_{2}=k_{b}+2 \\ 0 & otherwise \end{matrix} \right\}$ | (3) |
| --- | --- | --- |

Repeating this binomial sampling for each step, at generation $j$ the probability of observing $z$ additional individuals carrying minor alleles, for a total of $k_{b}+z$ minor alleles is:

|  | $P\left( k_{j}=k_{b}+z\vert k_{b} \right)=\left( \begin{matrix} j \\ z \end{matrix} \right)\left( \frac{\left( k_{b}+z-1 \right)!}{\left( k_{b}-1 \right)!} \right)\left( \frac{\left( \left( n_{b}-k_{b} \right)+\left( j-z-1 \right) \right)!}{\left( n_{b}-k_{b}-1 \right)!} \right)\left( \frac{\left( n_{b}-1 \right)!}{\left( n_{b}+\left( j-1 \right) \right)!} \right)$ | (4) |
| --- | --- | --- |

This closed-form equation (4) provides the probabilities for the transition matrix for moving from the initial $k_{b}$ minor alleles in the post-bottleneck population of size $n_{b}$, to the final number of alleles after genetic drift, $k_{j}$, in the final population of size $n_{b}+j$. The binomial sampling for the bottleneck in (1) and the genetic drift in equation (4) correspond to two transition matrices of a discrete Markov Chain.

*Modeling Sampling Error*

The next component of the full probability reflects the sampling process used to obtain the observed data set. Consider the true final minor allele count, $K_{F}$ in the full size final population of size $N_{F}$ and a sample of size $n_{F}$, the probability of observing $k_{F}$ ($0\leq k_{F}\leq n_{F}$) minor alleles in this sample follows a hypergeometric distribution, $k_{F}\sim Hypergeometric\left( n_{F}, K_{0}, N_{0} \right)$. As in the bottleneck process, $n_{F}<<N_{F}$, we use a binomial distribution to model this sampling, ($k_{F}\sim Binomial(n_{F},p_{F}=\frac{K_{F}}{N_{F}})$). Therefore, we calculate:

|  | $P\left( k_{F} \vert p_{F}, n_{F} \right)=\left( \begin{matrix} n_{F} \\ k_{F} \end{matrix} \right)p_{F}^{k_{F}}p_{F}^{n_{F}-k_{F}}$ | (5) |
| --- | --- | --- |

Additionally, we need to incorporate the sampling process for the initial population sample. In this case, the probability calculation is in the opposite direction and we aim to estimate $P(p_{0}|k_{I}, n_{I}$), the probability of the minor allele frequency in the initial full size population, $p_{0}$, given the minor allele count $k_{I}$ and sample size $n_{I}$. we apply Bayes’ Rule and the Total Probability Theorem, followed by the General Product Rule. The final reduction occurs because $n_{I}$ (the initial population sample size) is a known parameter and independent of $p_{0}$ ($P\left( n_{I} \right|p_{0})=P\left( n_{I} \right)=1)$. Now $P(k_{I}|p_{0},n_{I})$ is simply the sampling error described above in (5), the binomial probability of $k_{I}$ minor alleles in a sample of size $n_{I}$, drawn from the underlying frequency of $p_{0}$. We assume a uniform prior on $p_{0}$.

|  | $P\left( p_{0} \vert k_{I}, n_{I} \right)=\frac{P\left( k_{I}, n_{I} \vert p_{0} \right)P\left( p_{0} \right)}{\int_{0}^{1} \left[ P\left( k_{I}, n_{I} \vert p_{0} \right)P\left( p_{0} \right) \right]dp_{0}}=\frac{[P\left( k_{I} \vert p_{0}, n_{I} \right)P\left( n_{I} \vert p_{0} \right)]P(p_{0})}{\int_{0}^{1} \left[ P\left( k_{I} \vert p_{0}, n_{I} \right)P\left( n_{I} \vert p_{0} \right)P\left( p_{0} \right) \right]dp_{0}}=\frac{P\left( k_{I} \vert p_{0}, n_{I} \right)P(p_{0})}{\int_{0}^{1} \left[ P\left( k_{I} \vert p_{0}, n_{I} \right)P\left( p_{0} \right) \right]dp_{0}}$ | (6) |
| --- | --- | --- |

Modeling Genotyping Error

To model the genotyping errors, we incorporate a conservative position-specific error rate *ε* of 0.001 based on the base quality scores during sequencing. Consider the final sample of size $n_{F}$ with the true number of minor alleles $k_{F}$ ($0\leq k_{F}\leq n_{F}$), the probability that the number of observed minor alleles is $k_{F}^{obs}$ ($0\leq k_{F}^{obs} \leq n_{F}$) is determined by rate of errors in genotyping, $\varepsilon$. In (7), this probability is made up of two binomials that model: the probability that $i$ minor alleles are correctly called minor (with probability $(1-\varepsilon)$); and the probability that the remaining $k_{F}^{obs}-i$ alleles are incorrectly called minor (with probability $\varepsilon$).

|  | $P\left( k_{F}^{obs} \vert n_{F}, k_{F} \right)=\sum_{i=0}^{k_{F}^{obs}} \left( \begin{aligned} k_{F}^{obs} \\ i \end{aligned} \right)\left( 1-\varepsilon\right)^{i}\left( \varepsilon\right)^{k_{F}-i}\left( \begin{aligned} n_{F}-k_{F} \\ k_{F}^{obs} -i \end{aligned} \right)\varepsilon^{k_{F}^{obs} -i}\left( 1-\varepsilon\right)^{\left( n_{F}-k_{F} \right)-\left( k_{F}^{obs} -i \right)}$ | (7) |
| --- | --- | --- |

Because there is potential for genotyping error on both sides of the model (for the observed initial sample and the observed final sample), we also need to calculate the probability of the true initial minor allele count in the sample, $k_{I}$, given the observed value $k_{I}^{obs}$. To this end, we apply Bayes’ Rule and conditional probability to calculate $P\left( k_{I} \right|k_{I}^{obs}, n_{I})$ as shown in (8). We assume a uniform prior on $k_{I}$ so $P\left( k_{I} | n_{I} \right)/P\left( k_{I}^{obs} | n_{I} \right)$ is constant in $k_{I}.$ Therefore, this reduces to the genotyping error component as shown in (7), now for the initial population sample: the probability of observing $k_{I}^{obs}$ minor alleles in the initial sample of size $n_{I}$ given the true minor allele count of $k_{I}$.

|  | $P\left( k_{I} \right\vert k_{I}^{obs}, n_{I})=\frac{P\left( k_{I}^{obs}, k_{I} \vert n_{I} \right)}{P\left( k_{I}^{obs} \vert n_{I} \right)}=\frac{P\left( k_{I}^{obs} \vert n_{I},k_{I} \right)P(k_{I}\vert n_{I})}{P\left( k_{I}^{obs} \vert n_{I} \right)}\propto P\left( k_{I}^{obs} \vert n_{I},k_{I} \right)$ | (8) |
| --- | --- | --- |

*Combining Components for the Full Model*

With each of these components defined, we now return to the full probabilistic model, $P\left( k_{F}^{obs} \right|k_{I}^{obs}, n_{I}, n_{F}, n_{b})$. For reference, **Table A. 1** provides a list of the symbols and their meanings used throughout this derivation. First, we write $P\left( k_{F}^{obs} \right|k_{I}^{obs}, n_{I}, n_{F}, n_{b})$ by conditioning on $k_{b}$, the number of minor alleles in the bottleneck of size $n_{b}$:

|  | $P\left( k_{F}^{obs} \right\vert k_{I}^{obs}, n_{I}, n_{F}, n_{b})=\sum_{k_{0}=0}^{n_{0}} P\left( k_{F}^{obs} \right\vert k_{b}, k_{I}^{obs}, n_{I}, n_{F}, n_{b})P\left( k_{b} \vert k_{I}^{obs}, n_{I}, n_{F}, n_{b} \right)= \sum_{k_{b}=0}^{n_{b}} \underset{Part B}{\underbrace{P\left( k_{F}^{obs} \right\vert k_{b}, n_{F}, n_{b})}}\underset{Part A}{\underbrace{P\left( k_{b} \vert k_{I}^{obs}, n_{I}, n_{b} \right)}}$ | (9) |
| --- | --- | --- |

In (9), this probability further simplifies because $k_{F}^{obs}$ given $k_{b}, n_{F}, n_{b}$ is independent of $k_{I}^{obs}, n_{I}$ and $k_{b}$ given $k_{I}^{obs}, n_{I}, n_{b}$is independent of $n_{F}$. Therefore, this probability consists of two expressions: (A) the probability of transmitting $k_{b}$ minor alleles in a bottleneck of size $n_{b}$ given the initial observed minor allele frequency $k_{I}^{obs}$ and initial sample size $n_{I}$; and (B) the probability of observing $k_{F}^{obs}$ minor alleles after genetic drift, conditional on $k_{b}, n_{F}, n_{b}$. We will use conditional probabilities to break these parts into the interpretable components defined above.

To calculate Part A, we condition on $p_{0}$, the minor allele frequency in the initial population. Because $p_{0}$ is independent of $n_{b}$ given $k_{I}^{obs}, n_{I}$and because $k_{b}$ given $p_{0}, n_{b}$ is independent of $k_{I}^{obs}, n_{I}$ and $p_{0}$, this simplifies to two additional parts in (10).

|  | $P\left( k_{0} \vert k_{I}^{obs}, n_{I}, n_{0} \right)=\int_{0}^{1} P\left( k_{0}\vert p_{0}, {k_{I}^{obs}, n_{I}, n}_{0} \right)P\left( p_{0}\vert k_{I}^{obs}, n_{I}, n_{0} \right)dp_{0}$  $=\int_{0}^{1} \underset{A_{1}}{\underbrace{P\left( k_{0}\vert p_{0}, n_{0} \right)}}\underset{A_{2}}{\underbrace{P\left( p_{0}\vert k_{I}^{obs}, n_{I} \right)}}dp_{0}$ | (10) |
| --- | --- | --- |

The first part of this expression, A_1_, is simply the bottleneck step as in (1).

The second expression of this equation, A_2_, requires further work. We first condition on $k_{I},$the true minor allele count and A_2_ simplifies to (11) since $p_{0}$ given $n_{I}, k_{I}$is independent of $k_{I}^{obs}$. This expression now includes the genotyping and sampling processes for the initial population sample as outlined in previous sections.

|  | $P\left( p_{0}\vert k_{I}^{obs}, n_{I} \right)=\sum_{k_{I}=0}^{n_{I}} P\left( p_{0} \right\vert k_{I}^{obs}, n_{I}, k_{I})P\left( k_{I} \right\vert k_{I}^{obs}, n_{I})=\sum_{k_{I}=0}^{n_{I}} P(p_{0}\vert n_{I}, k_{I})P\left( k_{I} \right\vert k_{I}^{obs}, n_{I})$ | (11) |
| --- | --- | --- |

In (12), we incorporate the sampling error as calculated in (6), and the genotyping error as calculated in (8).

|  | $\sum_{k_{I}=0}^{n_{I}} P(p_{0}\vert n_{I}, k_{I})P\left( k_{I} \right\vert k_{I}^{obs}, n_{I})\propto\sum_{k_{I}=0}^{n_{I}} \left\{ \left( \frac{P\left( k_{I} \vert p_{0}, n_{I} \right)P(p_{0})}{\int_{0}^{1} P\left( k_{I} \vert p_{0}, n_{I} \right)P(p_{0})dp_{0}} \right)P\left( k_{I}^{obs} \vert{k_{I}, n}_{I} \right) \right\}$ | (12) |
| --- | --- | --- |

We now focus on Part B of (9), the probability of observing $k_{F}^{obs}$ minor alleles after genetic drift, conditional on $k_{b}, n_{b},$and $n_{F}$. This part models the three processes that occur after the bottleneck: (1) genetic drift (growth) to reach the final minor allele count $K_{F}$ and final total allele count $N_{F}$ from the bottleneck size of $n_{I}$, (2) sampling from this final population, (3) genotyping error in our sample. To reach these interpretable parts, we start by conditioning on $k_{F}$, the true minor allele count in the sample, and then $K_{F}$, the final population minor allele count.

|  | $P\left( k_{F}^{obs} \right\vert k_{b},n_{b}, n_{F})=\sum_{k_{F}=0}^{n_{F}} P\left( k_{F}^{obs}\vert k_{F},n_{b},k_{b}, n_{F} \right)P\left( k_{F} \vert k_{b},n_{b}, n_{F} \right)$  $=\sum_{k_{F}=0}^{n_{F}} P\left( k_{F}^{obs}\vert k_{F}, n_{F} \right)P\left( k_{F} \vert k_{b},n_{b}, n_{F} \right)=$ $\sum_{k_{F}=0}^{n_{F}} \left[ P\left( k_{F}^{obs}\vert k_{F}, n_{F} \right)\sum_{K_{F}=0}^{N_{F}=0} P\left( k_{F} \vert k_{b},n_{b}, n_{F}, K_{F}, N_{F} \right)P\left( K_{F} \vert N_{F}, k_{b},n_{b}, n_{F} \right) \right]=\sum_{k_{F}=0}^{n_{F}} \left[ \underset{B_{1}}{\underbrace{P\left( k_{F}^{obs}\vert k_{F}, n_{F} \right)}}\sum_{K_{F}=0}^{N_{F}=0} \underset{B_{2}}{\underbrace{P\left( k_{F} \vert n_{F}, K_{F}, N_{F} \right)}}\underset{B_{3}}{\underbrace{P\left( K_{F} \vert N_{F}, k_{b},n_{b} \right)}} \right]$ | (13) |
| --- | --- | --- |

The first expression in (13), B_1_, arises because $k_{F}^{obs}$ is independent of $n_{b},k_{b}$ given $k_{F}, n_{F}$. Then B_1_ is the genotyping error probability, as calculated in (7).

The second term, B_2_, arises by conditioning on $K_{F}, N_{F},$ such that $P\left( k_{F} | n_{F}, K_{F}, N_{F} \right)$ is independent of $k_{b}, n_{b}$ (we assume $N_{F}$, the final population size, is known). Then B_2_ is the sampling error component as in (5): a binomial that corresponds to the observing $k_{F}$ minor alleles after sampling $n_{F}$ from the full final population where minor alleles are sampled with probability $p_{F}=K_{F}/N_{F}$.

The last portion, B_3_, models the growth of the population (genetic drift) to the full size final population from the bottleneck size. In B_3_, $K_{F}$ is independent of$n_{F}$ given $k_{b},n_{b},N_{F}$. As in (4), B_3_ is calculated using a modified Moran model without replacement, with $K_{F}=k_{b}+j$ and $N_{F}=n_{b}+j$.

Combining these components gives the overall summation in (9). This is the basic model for the bottleneck and subsequent growth.

*Calculating P-Value for Testing the Null Hypothesis of Genetic Drift*

We aim to test the null hypothesis of the change in minor allele frequency of a site between the initial to the final population under a model of genetic drift acting alone. To this end, we calculate a p-value: the probability, given the early stage minor allele count, of observing a late stage minor allele count at least as extreme as that observed in the data, under the null model of basic genetic drift. Using the notation of the mathematical model described above, we calculate the probability of the observed final allele count $k_{F}^{obs}$ given $n_{b}$, $n_{F}, k_{I}^{obs},$ and $n_{I}$ as in (9): $P(k_{F}^{obs}|n_{b}, n_{F}, k_{I}^{obs}, n_{I})$. To test the null hypothesis of genetic drift alone, we calculate the individual probabilities of observing each minor allele count at least as extreme as $\hat{k}_{F}$. For example, if the final minor allele count is greater than the initial minor allele count, indicating the minor allele count has increased, we calculate each $k_{F}^{*}\geq k_{F}^{obs}$. Summing over these probabilities, we obtain $P\left( k_{F}^{*}\geq k_{F}^{obs} | n_{0}, n_{F}, k_{I}^{obs}, n_{I} \right)=\sum_{k_{F}^{*}=k_{F}^{obs}}^{k_{F}^{*}=n_{F}} P\left( k_{F}^{*} | n_{0}, n_{F}, k_{I}^{obs}, n_{I} \right)$. Similarly, if the observed final minor allele count is less than the initial minor allele count, indicating the minor allele count has decreased, we calculate $P\left( k_{F}^{*}\leq k_{F}^{obs} | n_{0}, n_{F}, k_{I}^{obs}, n_{I} \right)=\sum_{k_{F}^{*}=0}^{k_{F}^{*}=\hat{k}_{F}} P\left( k_{F}^{*} | n_{0}, n_{F}, k_{I}^{obs}, n_{I} \right)$. This formulation provides a closed-form equation to calculate a p-value: the probability of change in allele frequency at least as extreme as that observed, under the null hypothesis of genetic drift alone. Where this p-value is sufficiently small, we have evidence against this null hypothesis. Because a total of 290kb genomic region was chosen for the targeted sequencing and further assessed for identification of possible somatic mutations, we adjust for multiple testing by comparing each p-value to a Bonferroni corrected alpha (α=0.05/2.9x10^-7^= 1.7 x 10^-7^).

*Estimating Selection Coefficients*

Where there is significant evidence against the null hypothesis of drift alone, we estimate $s$, the selection coefficient of the variant. The selection coefficient is a measure of the relative fitness of individuals carrying the minor allele^3^. Individuals carrying the minor allele have an increased probability of reproducing by a factor of $(1+s)$. We construct a grid of possible values for $s$: (-1.0, -0.9, -0.8, -0.7, -0.6, -0.5, -0.4, -0.3, -0.25, -0.2, -0.15, -0.1, -0.05, -0.01, 0.0, 0.01, 0.05, 0.1, 0.15, 0.2, 0.25, 0.3, 0.4, 0.5, 0.6, 0.7, 0.8, 0.9, 1.0) based on the changes in minor allele count in the data set. We use an upper limit of $s=1.0$ (indicating very strong selection and increasing the probability of reproduction by a factor of 2) because a larger $s$ is unlikely when the observed final minor allele frequencies do not approach 1.0. We have additional points between -0.25 and 0.25 to increase precision where we anticipated the most likely estimates of $s$. For each possible value for $s$in the grid, we calculate $L\left( s| n_{0},k_{F}^{obs}, n_{F}, k_{I}^{obs}, n_{I} \right)$, the likelihood of this $s$ given the observed data. We choose this grid with an additional points in Maintaining the basic model above, we adjust the step-wise probabilities (2) such that individuals carrying the minor allele have an increased probability of being selected for replication of $(1+s).$ After normalization, the probability of $k_{1}$ minor alleles in the next generation, given $k_{b}$ in the current population of size $n_{b}$ is:

|  | $P\left( k_{1} \right\vert n_{b}, k_{b})= \left\{ \begin{matrix} \frac{n_{b}-k_{b}}{n_{b}+k_{b}s} & if k_{1}=k_{b} \\ \frac{k_{b}+k_{b}s}{n_{b}+k_{b}s} & if k_{1}=k_{b}+1 \\ 0 & otherwise \end{matrix} \right\}$ | (14) |
| --- | --- | --- |

Under this formulation, the closed-form expression of (4) is no longer available. Therefore, to obtain Part B_3_ in equation (12), we apply Monte Carlo integration based on 10,000 random walks using these step-wise probabilities from the post-bottleneck population size, $n_{0}=200$, to the final population size, $N_{F}$. For each walk, we record the simulate final minor allele frequency, $K_{F}$, producing a probability distribution over $K_{F}$to be used for Part B_3_. The rest of the model remains unchanged. The $s$ with the maximum likelihood across this grid is the maximum likelihood estimate (MLE) of the selection coefficient. In addition, we calculate the acceptance region of the MLE using the log-likelihood ratio test with a chi-square value of one degree of freedom. The grid values whose likelihoods are contained in this region make up the reported 95% confidence interval.

1. Casella, G., and Berger, R.L. (2002). Statistical Inference.(Thomson Learning).

2. Moran, P.A.P. (1962). The statistical processes of evolutionary theory.(Oxford,: Clarendon Press).

3. Gillespie, J.H. (1998). Population genetics : a concise guide.(Baltimore, Md: The Johns Hopkins University Press).
